# Dibromido[bis­(η^5^-cyclo­penta­dien­yl)dimethyl­silane]zirconium(IV)

**DOI:** 10.1107/S1600536808040713

**Published:** 2008-12-17

**Authors:** Milan Erben, Michal Dušek, Michal Picka, Jaromír Vinklárek

**Affiliations:** a Department of General and Inorganic Chemistry, Faculty of Chemical Technology, University of Pardubice, Nám. Čs. legií 565, Pardubice 532 10, Czech Republic

## Abstract

The title mol­ecule, [ZrBr_2_(C_12_H_14_Si)], possesses a crystallographically imposed twofold rotational symmetry with the rotation axis passing through the Zr and Si atoms. The Zr^IV^ centre is in a distorted tetra­hedral environment defined by two Cp rings of chelating organic ligands and two Br anions. Two five-membered rings form a dihedral angle of 59.7 (2)°. Unequal Zr—C bonds [2.471 (3)–2.556 (3) Å] in the mol­ecule indicate that the inter­action of the central metal with the [(C_5_H_4_)_2_SiMe_2_]^2−^ ligand contains noticeable η^3^-allyl and η^2^-olefin contributions.

## Related literature

For related ansa-zirconocenes, see, for example: Bajgur *et al.* (1985 ▶), Borrelli *et al.* (2002 ▶).
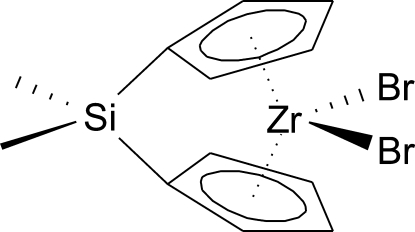


## Experimental

### 

#### Crystal data

[ZrBr_2_(C_12_H_14_Si)]
*M* *_r_* = 437.36
Monoclinic, 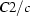

*a* = 13.6160 (4) Å
*b* = 10.0990 (2) Å
*c* = 10.9770 (3) Å
β = 112.2540 (12)°
*V* = 1396.99 (6) Å^3^
*Z* = 4
Mo *K*α radiation
μ = 6.57 mm^−1^
*T* = 120 (2) K
0.40 × 0.30 × 0.22 mm

#### Data collection

Nonius KappaCCD area-detector diffractometer
Absorption correction: Gaussian (Coppens *et al*., 1970 ▶) *T*_min_ = 0.137, *T*_max_ = 0.271
9698 measured reflections
1602 independent reflections
1560 reflections with *I* > 2σ(*I*)
*R*_int_ = 0.047

#### Refinement

*R*[*F* ^2^ > 2σ(*F* ^2^)] = 0.024
*wR*(*F* ^2^) = 0.063
*S* = 1.14
1602 reflections
76 parameters
H-atom parameters constrained
Δρ_max_ = 0.69 e Å^−3^
Δρ_min_ = −1.07 e Å^−3^

### 

Data collection: *COLLECT* (Hooft, 1998 ▶) and *DENZO* (Otwin­owski & Minor, 1997 ▶); cell refinement: *COLLECT* and *DENZO*; data reduction: *COLLECT* and *DENZO*; program(s) used to solve structure: *SIR92* (Altomare *et al.*, 1994 ▶); program(s) used to refine structure: *SHELXL97* (Sheldrick, 2008 ▶); molecular graphics: *PLATON* (Spek, 2003 ▶); software used to prepare material for publication: *enCIFer* (Allen *et al.*, 2004 ▶).

## Supplementary Material

Crystal structure: contains datablocks I, global. DOI: 10.1107/S1600536808040713/cv2494sup1.cif

Structure factors: contains datablocks I. DOI: 10.1107/S1600536808040713/cv2494Isup2.hkl

Additional supplementary materials:  crystallographic information; 3D view; checkCIF report

## Figures and Tables

**Table 1:** Selected geometric parameters (Å°, )

Zr1—*Cg*1	2.199 (1)	C6—Si1—C6^i^	116.0 (1)
Zr1—Br1	2.6007 (4)	C1—Si1—C1^i^	93.2 (1)
*Cg*1—Zr1—*Cg*1^i^	125.96 (5)	Br1—Zr1—Br1^i^	98.39 (1)

Symmetry code: (i) 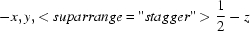. *Cg*1 is the centroid of atoms C1–C5.

**Fig. 1.**
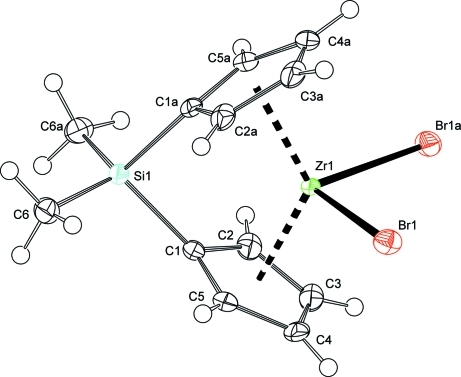
Perspective view of (I), shown with 30% probability displacement ellipsoids (arbitrary spheres for H atoms) [symmetry code: (a) -x, y, 1/2-z.]

## supplementary crystallographic information

### Comment

Metallocene complexes of Group 4 are intensively investigated as very efficient and selective olefin polymerization and co-polymerization catalysts (Borrelli *et al.*, 2002). The most used strategy for the modification of catalytic properties of these metallocenes is the substitution at the cyclopentadienyl rings. Incorporation of an interannular bridge connecting both cyclopentadienyl rings in the molecule of metallocene leads to a broad class of complexes known as ansa-metallocenes. As part of an investigation of the influence of ring substitution on the properties of cyclopentadienyl complexes, the title compound, (I), was prepared, spectroscopically characterized and its structure determined

A perspective view of molecular structure of (I) is shown in Figure 1 with appropriate atom labeling scheme, selected bond distances and angles are summarized in Table 1. In the molecule of (I) the Zr atom is pseudotetrahedrally coordinated by two η^5^-bonded Cp rings and two Br atoms with geometry constrained by crystallographic twofold rotation axis, which bisects Br1—Zr—Br1a angle and passes through the metal and Si atom. The molecular parameters are comparable to those reported for [ZrCl_2_(η^5^-C_5_H_4_)_2_SiMe_2_] (Bajgur *et al.*, 1985). On the inspection of parameters associated with Cp rings, the deviation from the ideal η^5^-bonding fashion could be observed. The C3—C4 bond distance of 1.400 (6) Å is shorter than the remaining C—C bonds (average of 1.42 Å). Similarly Zr1—C3 and Zr1—C4 bonds are longer [2.556 (3) Å and 2.552 (4) Å, respectively] than remaining metal-carbon bonds (average of 2.475 Å). These facts indicate the presence of η^3^-allyl and η^2^-olefin bonding fashion of Cp ring rather than η^5^-cyclopentadienyl bonding pattern.

### Experimental

Compound (I) was prepared by bromination of analogous chloride derivative using boron tribromide. To the starting complex [ZrCl_2_(η^5^-C_5_H_4_)_2_SiMe_2_] (0.2 g; 0.57 mol) in 20 ml of dichloromethane 0.04 ml (0.42 mmol) of BBr_3_ was added. The color of the reaction mixture immediatelly turned to green and it was stirred for additional 2 h at 293 K. The solvent was evaporated in vacuum, solid residue was washed with hexane (2x5 ml) and vacuum-dried. Sublimation of crude product at 10 ^-3^ Pa and 475 K gave 0.105 g (42%) of (I). Crystals of (I) suitable for X-ray diffraction measurements were grown during slow evaporation of chloroform solution at 273 K.

### Refinement

All H atoms were positioned geometrically and refined as riding on their parent C atoms, with C—H = 0.93 Å, *U*_iso_(H) = 1.2U*_e.g_*(C) and C—H = 0.96 Å, *U*_iso_(H) = 1.5U*_e.g_*(C) for cyclopentadienyl and methyl H atoms, respectively.

### Crystal data

**Table:**

[ZrBr_2_(C_12_H_14_Si)]	*F*(000) = 840
*M**_r_* = 437.36	*D*_x_ = 2.079 Mg m^−^^3^
Monoclinic, *C*2/*c*	Mo *K*α radiation, λ = 0.71073 Å
Hall symbol: -C 2yc	Cell parameters from 6471 reflections
*a* = 13.6160 (4) Å	θ = 1–27.5°
*b* = 10.0990 (2) Å	µ = 6.57 mm^−^^1^
*c* = 10.9770 (3) Å	*T* = 120 K
β = 112.2540 (12)°	Prism, yellow
*V* = 1396.99 (6) Å^3^	0.4 × 0.3 × 0.22 mm
*Z* = 4	

### Data collection

**Table:**

Nonius KappaCCD area-detector diffractometer	1602 independent reflections
Radiation source: fine-focus sealed tube	1560 reflections with *I* > 2σ(*I*)
graphite	*R*_int_ = 0.047
Detector resolution: 9.091 pixels mm^-1^	θ_max_ = 27.5°, θ_min_ = 2.6°
φ and ω scans to fill the Ewald sphere	*h* = −17→17
Absorption correction: gaussian (Coppens et al., 1970)	*k* = −13→13
*T*_min_ = 0.137, *T*_max_ = 0.271	*l* = −14→14
9698 measured reflections	

### Refinement

**Table:**

Refinement on *F*^2^	Secondary atom site location: difference Fourier map
Least-squares matrix: full	Hydrogen site location: inferred from neighbouring sites
*R*[*F*^2^ > 2σ(*F*^2^)] = 0.024	H-atom parameters constrained
*wR*(*F*^2^) = 0.063	*w* = 1/[σ^2^(*F*_o_^2^) + (0.0323*P*)^2^ + 3.2435*P*] where *P* = (*F*_o_^2^ + 2*F*_c_^2^)/3
*S* = 1.14	(Δ/σ)_max_ < 0.001
1602 reflections	Δρ_max_ = 0.69 e Å^−^^3^
76 parameters	Δρ_min_ = −1.07 e Å^−^^3^
0 restraints	Extinction correction: *SHELXL97* (Sheldrick, 1997), Fc^*^=kFc[1+0.001xFc^2^λ^3^/sin(2θ)]^-1/4^
Primary atom site location: structure-invariant direct methods	Extinction coefficient: 0.0044 (3)

### Special details

**Table:**

Experimental. Spectroscopic analysis: ^1^H NMR (CDCl_3_, δ, p.p.m.): 7.15 (m, 4H), 5.97 (m, 4H), 0.73 (s, 6H). ^13^C NMR (CDCl_3_, δ, p.p.m.): -5.2, 1, 114.3, 115.8, 128.9. IR (KBr disc, cm^-1^): 3115 (*m*), 3099 (*m*), 3074 (*m*), 2964 (*m*), 2958 (*m*), 1448 (*m*), 1409 (w), 1400 (*s*), 1370 (*s*), 1362 (*m*), 1324 (*m*), 1314 (w), 1260 (*s*), 1200 (w), 1179 (*s*), 1168 (*s*), 1069 (*s*), 1048 (*s*), 1020 (*s*), 942 (*m*), 902 (*s*), 870 (*s*), 870 (*s*), 813 (*s*), 680 (*s*), 633 (*s*), 555 (*m*), 502 (*m*), 454 (*s*), 420 (*s*), 384 (*m*), 347 (*s*); UV-Vis (CH_2_Cl_2_, maxima at nm): 470, 377, 313, 233. Elemental analysis, calculated for C_12_H_14_Br_2_SiZr: C 32.96, H 3.23; found: C 32.61, H 3.14%.
Geometry. All e.s.d.'s (except the e.s.d. in the dihedral angle between two l.s. planes) are estimated using the full covariance matrix. The cell e.s.d.'s are taken into account individually in the estimation of e.s.d.'s in distances, angles and torsion angles; correlations between e.s.d.'s in cell parameters are only used when they are defined by crystal symmetry. An approximate (isotropic) treatment of cell e.s.d.'s is used for estimating e.s.d.'s involving l.s. planes.
Refinement. Refinement of *F*^2^ against ALL reflections. The weighted *R*-factor *wR* and goodness of fit *S* are based on *F*^2^, conventional *R*-factors *R* are based on *F*, with *F* set to zero for negative *F*^2^. The threshold expression of *F*^2^ > σ(*F*^2^) is used only for calculating *R*-factors(gt) *etc*. and is not relevant to the choice of reflections for refinement. *R*-factors based on *F*^2^ are statistically about twice as large as those based on *F*, and *R*- factors based on ALL data will be even larger.

### Fractional atomic coordinates and isotropic or equivalent isotropic displacement parameters (Å^2^)

**Table:**

	*x*	*y*	*z*	*U*_iso_*/*U*_eq_	
Zr1	0.0000	0.31648 (3)	0.2500	0.01164 (12)	
Br1	0.155827 (19)	0.48477 (3)	0.33572 (2)	0.02090 (12)	
Si1	0.0000	−0.01625 (9)	0.2500	0.0160 (2)	
C1	0.0077 (2)	0.1116 (2)	0.1295 (2)	0.0170 (5)	
C2	−0.0792 (2)	0.1870 (3)	0.0443 (3)	0.0206 (5)	
H2	−0.1504	0.1647	0.0193	0.025*	
C3	−0.0400 (3)	0.3017 (3)	0.0033 (3)	0.0250 (6)	
H3	−0.0807	0.3658	−0.0547	0.030*	
C4	0.0707 (3)	0.3014 (3)	0.0658 (3)	0.0247 (6)	
H4	0.1166	0.3654	0.0567	0.030*	
C5	0.1003 (2)	0.1861 (3)	0.1454 (3)	0.0207 (5)	
H5	0.1693	0.1630	0.1993	0.025*	
C6	0.1245 (2)	−0.1135 (3)	0.3168 (3)	0.0247 (6)	
H6A	0.1191	−0.1775	0.3787	0.037*	
H6B	0.1830	−0.0551	0.3603	0.037*	
H6C	0.1359	−0.1583	0.2462	0.037*	

### Atomic displacement parameters (Å^2^)

**Table:**

	*U*^11^	*U*^22^	*U*^33^	*U*^12^	*U*^13^	*U*^23^
Zr1	0.01466 (17)	0.01152 (18)	0.01051 (17)	0.000	0.00678 (12)	0.000
Br1	0.01687 (16)	0.02362 (18)	0.02273 (17)	−0.00544 (9)	0.00811 (11)	−0.00413 (9)
Si1	0.0237 (5)	0.0120 (4)	0.0156 (5)	0.000	0.0112 (4)	0.000
C1	0.0261 (12)	0.0145 (11)	0.0147 (11)	0.0005 (10)	0.0125 (9)	−0.0021 (9)
C2	0.0282 (13)	0.0187 (13)	0.0134 (11)	−0.0003 (10)	0.0062 (10)	−0.0028 (9)
C3	0.0467 (17)	0.0181 (13)	0.0122 (11)	0.0029 (12)	0.0134 (11)	0.0003 (10)
C4	0.0435 (16)	0.0189 (13)	0.0224 (13)	−0.0035 (12)	0.0245 (12)	−0.0017 (10)
C5	0.0272 (13)	0.0193 (13)	0.0227 (13)	0.0015 (10)	0.0176 (11)	−0.0012 (10)
C6	0.0321 (14)	0.0228 (14)	0.0220 (13)	0.0080 (12)	0.0135 (11)	0.0048 (11)

### Geometric parameters (Å, °)

**Table:**

Zr1—C5^i^	2.471 (3)	Si1—C1^i^	1.880 (3)
Zr1—C5	2.471 (3)	Si1—C1	1.880 (3)
Zr1—C2^i^	2.476 (3)	C1—C2	1.420 (4)
Zr1—C2	2.476 (3)	C1—C5	1.422 (4)
Zr1—C1	2.480 (2)	C2—C3	1.418 (4)
Zr1—C1^i^	2.480 (2)	C2—H2	0.9300
Zr1—C4^i^	2.552 (3)	C3—C4	1.400 (4)
Zr1—C4	2.552 (3)	C3—H3	0.9300
Zr1—C3	2.556 (3)	C4—C5	1.420 (4)
Zr1—C3^i^	2.556 (3)	C4—H4	0.9300
Zr1—Br1^i^	2.6007 (3)	C5—H5	0.9300
Zr1—Br1	2.6007 (3)	C6—H6A	0.9600
Si1—C6^i^	1.853 (3)	C6—H6B	0.9600
Si1—C6	1.853 (3)	C6—H6C	0.9600
			
C5^i^—Zr1—C5	115.60 (12)	C3^i^—Zr1—Br1^i^	103.82 (7)
C5^i^—Zr1—C2^i^	54.64 (9)	C5^i^—Zr1—Br1	134.35 (7)
C5—Zr1—C2^i^	90.90 (9)	C5—Zr1—Br1	89.86 (6)
C5^i^—Zr1—C2	90.90 (9)	C2^i^—Zr1—Br1	90.07 (6)
C5—Zr1—C2	54.64 (9)	C2—Zr1—Br1	133.57 (7)
C2^i^—Zr1—C2	116.22 (12)	C1—Zr1—Br1	123.01 (6)
C5^i^—Zr1—C1	86.89 (8)	C1^i^—Zr1—Br1	123.08 (6)
C5—Zr1—C1	33.37 (8)	C4^i^—Zr1—Br1	104.65 (7)
C2^i^—Zr1—C1	87.36 (9)	C4—Zr1—Br1	79.92 (7)
C2—Zr1—C1	33.31 (9)	C3—Zr1—Br1	103.82 (7)
C5^i^—Zr1—C1^i^	33.37 (8)	C3^i^—Zr1—Br1	80.64 (7)
C5—Zr1—C1^i^	86.89 (8)	Br1^i^—Zr1—Br1	98.387 (17)
C2^i^—Zr1—C1^i^	33.31 (9)	C6^i^—Si1—C6	116.0 (2)
C2—Zr1—C1^i^	87.36 (9)	C6^i^—Si1—C1^i^	110.88 (12)
C1—Zr1—C1^i^	66.87 (11)	C6—Si1—C1^i^	111.83 (12)
C5^i^—Zr1—C4^i^	32.79 (9)	C6^i^—Si1—C1	111.83 (12)
C5—Zr1—C4^i^	141.00 (9)	C6—Si1—C1	110.87 (12)
C2^i^—Zr1—C4^i^	53.88 (9)	C1^i^—Si1—C1	93.26 (15)
C2—Zr1—C4^i^	121.77 (9)	C2—C1—C5	106.1 (2)
C1—Zr1—C4^i^	118.55 (9)	C2—C1—Si1	125.24 (19)
C1^i^—Zr1—C4^i^	54.73 (8)	C5—C1—Si1	124.1 (2)
C5^i^—Zr1—C4	141.00 (9)	C2—C1—Zr1	73.19 (14)
C5—Zr1—C4	32.80 (9)	C5—C1—Zr1	72.99 (14)
C2^i^—Zr1—C4	121.77 (9)	Si1—C1—Zr1	99.94 (10)
C2—Zr1—C4	53.88 (9)	C3—C2—C1	109.1 (2)
C1—Zr1—C4	54.73 (8)	C3—C2—Zr1	76.77 (15)
C1^i^—Zr1—C4	118.55 (9)	C1—C2—Zr1	73.51 (14)
C4^i^—Zr1—C4	173.17 (13)	C3—C2—H2	125.5
C5^i^—Zr1—C3	121.83 (10)	C1—C2—H2	125.5
C5—Zr1—C3	53.87 (9)	Zr1—C2—H2	116.2
C2^i^—Zr1—C3	141.26 (9)	C4—C3—C2	107.9 (2)
C2—Zr1—C3	32.69 (9)	C4—C3—Zr1	73.94 (15)
C1—Zr1—C3	54.63 (8)	C2—C3—Zr1	70.54 (15)
C1^i^—Zr1—C3	118.78 (9)	C4—C3—H3	126.0
C4^i^—Zr1—C3	147.45 (10)	C2—C3—H3	126.0
C4—Zr1—C3	31.81 (10)	Zr1—C3—H3	121.2
C5^i^—Zr1—C3^i^	53.87 (9)	C3—C4—C5	107.8 (2)
C5—Zr1—C3^i^	121.83 (10)	C3—C4—Zr1	74.25 (15)
C2^i^—Zr1—C3^i^	32.69 (9)	C5—C4—Zr1	70.48 (14)
C2—Zr1—C3^i^	141.25 (9)	C3—C4—H4	126.1
C1—Zr1—C3^i^	118.78 (9)	C5—C4—H4	126.1
C1^i^—Zr1—C3^i^	54.63 (8)	Zr1—C4—H4	120.9
C4^i^—Zr1—C3^i^	31.81 (10)	C4—C5—C1	109.0 (2)
C4—Zr1—C3^i^	147.45 (10)	C4—C5—Zr1	76.72 (15)
C3—Zr1—C3^i^	173.31 (13)	C1—C5—Zr1	73.64 (14)
C5^i^—Zr1—Br1^i^	89.86 (6)	C4—C5—H5	125.5
C5—Zr1—Br1^i^	134.35 (7)	C1—C5—H5	125.5
C2^i^—Zr1—Br1^i^	133.57 (7)	Zr1—C5—H5	116.1
C2—Zr1—Br1^i^	90.07 (6)	Si1—C6—H6A	109.5
C1—Zr1—Br1^i^	123.08 (6)	Si1—C6—H6B	109.5
C1^i^—Zr1—Br1^i^	123.01 (6)	H6A—C6—H6B	109.5
C4^i^—Zr1—Br1^i^	79.92 (7)	Si1—C6—H6C	109.5
C4—Zr1—Br1^i^	104.65 (7)	H6A—C6—H6C	109.5
C3—Zr1—Br1^i^	80.64 (7)	H6B—C6—H6C	109.5

Symmetry codes: (i) −*x*, *y*, −*z*+1/2.

### Table 1 Selected geometric parameters (Å, °)

**Table:**

Zr1—Cg1	2.199 (1)	C6—Si1—C6^i^	116.0 (1)
Zr1—Br1	2.6007 (4)	C1—Si1—C1^i^	93.2 (1)
Cg1—Zr1—Cg1^i^	125.96 (5)	Br1—Zr1—Br1^i^	98.39 (1)

Symmetry code: (i) -x, y, 1/2-z. Cg1 is the centroid of atoms C1–C5.

## References

[bb1] Allen, F. H., Johnson, O., Shields, G. P., Smith, B. R. & Towler, M. (2004). *J. Appl. Cryst.* **37**, 335–338.

[bb2] Altomare, A., Cascarano, G., Giacovazzo, C., Guagliardi, A., Burla, M. C., Polidori, G. & Camalli, M. (1994). *J. Appl. Cryst.* **27**, 435.

[bb3] Bajgur, C. S., Tikkanen, W. R. & Petersen, J. L. (1985). *Inorg. Chem.* **24**, 2539–2546.

[bb4] Borrelli, M., Busico, V., Cipulo, R. & Ronca, S. (2002). *Macromolecules*, **35**, 2835–2844.

[bb5] Coppens, P. (1970). *Crystallographic Computing*, edited by F. R. Ahmed, S. R. Hall & C. P. Huber, pp. 255–270. Copenhagen: Munksgaard.

[bb6] Hooft, R. W. (1998). *COLLECT* Nonius BV, Delft, The Netherlands.

[bb7] Otwinowski, Z. & Minor, W. (1997). *Methods in Enzymology*, Vol. 276, *Macromolecular Crystallography*, Part A, edited by C. W. Carter Jr & R. M. Sweet, pp. 307–326. New York: Academic Press.

[bb8] Sheldrick, G. M. (2008). *Acta Cryst.* A**64**, 112–122. 10.1107/S0108767307043930 18156677

[bb9] Spek, A. L. (2003). *J. Appl. Cryst.* **36**, 7–13.

